# Age- and Sex-Specific Reference Values for Renal Volume and Association with Risk Factors for Chronic Kidney Disease in a General Population—An MRI-Based Study

**DOI:** 10.3390/jcm13030769

**Published:** 2024-01-29

**Authors:** Thomas Dabers, Peter Sass, Fritz Fechner, Julian Weyer, Henry Völzke, Andreas Horst Mahnken, Roberto Lorbeer, Birger Mensel, Sylvia Stracke

**Affiliations:** 1 Nephrology, Internal Medicine A, University Medicine Greifswald, 17475 Greifswald, Germany; thomas.dabers@med.uni-greifswald.de (T.D.);; 2 KfH Renal Center, 17475 Greifswald, Germany; 3 Institute for Community Medicine—SHIP Clinical-Epidemiological Research, University Medicine Greifswald, 17475 Greifswald, Germany; voelzke@uni-greifswald.de; 4 Department of Diagnostic & Interventional Radiology, Philipps-University Marburg, 35037 Marburg, Germany; andreas.mahnken@staff.uni-marburg.de; 5 Department of Radiology, University Hospital, LMU Munich, 80539 Munich, Germany; roberto.lorbeer@med.uni-muenchen.de; 6 Department of Diagnostic and Interventional Radiology and Neuroradiology, Central Hospital Bad Berka, 99438 Bad Berka, Germany

**Keywords:** renal volume, CKD, magnetic resonance imaging, population-based studies

## Abstract

Background: Renal volume (RV) is associated with renal function and with a variety of cardiovascular risk factors (CVRFs). We analysed RV using magnetic resonance imaging (MRI) in a large population-based study (Study of Health in Pomerania; SHIP-TREND) to find sex- and age-specific reference values for RV and to test the influence of several markers on RV. The main objective is to describe reference values for RV in people from the general population without kidney disease. Methods: 1815 participants without kidney disease (930 women) aged 21–81 years were included in our study. Right and left RV with and without body surface area (BSA) indexation were compared among three age groups (22–39 years, 40–59 years, 60–81 years) by median and interquartile range and tested separately in women and men. Results: The estimated glomerular filtration rate (eGFR), serum uric acid, and right and left RV were higher in men compared to women (all *p* < 0.001). Left kidneys were larger than right kidneys (both sexes). With age, RV showed a continuously decreasing trend in women and an upside-down U-shaped relation in men. In multivariable linear regression models, current smoking (β = 14.96, 95% CI 12.12; 17.79), BSA (β = 97.66, 95% CI 90.4; 104.93), diastolic blood pressure (β = 0.17, 95% CI 0.01; 0.32), and eGFR (β = 0.57, 95% CI 0.50; 0.65) were positively associated with both left and right RV, whereas uric acid (β = −0.03, 95% CI −0.05; −0.01) showed an inverse association with RV. Interestingly, the same eGFR correlated with higher RV in men compared to women. Conclusion: Reference values for RV are different for age groups and sex. For any given age, female kidneys are smaller than male kidneys. RV associates positively with eGFR, but for any chosen eGFR, renal volume in females is lower compared to males. RV decreases with age, but in men showed a U-shaped correlation. This may reflect hyperfiltration and glomerular hypertrophy associated with the presence of CVRF in middle-aged males.

## 1. Introduction

Kidney size and length, as well as renal parenchyma thickness, are associated with renal function and with a variety of cardiovascular risk factors [1,2]. Renal imaging is used to detect and follow-up chronic kidney disease (CKD), kidney transplants, and renal artery stenosis [1,3,4,5]. Although ultrasound (US) is the more commonly used procedure, due to its accessibility in routine clinical practice and real-time imaging capabilities, magnetic resonance imaging (MRI) is another valid and reliable tool for measuring renal volume (RV) [6,7]. MRI is an accepted method for multiplanar imaging and provides superior soft-tissue contrast [2]. To establish the MRI measurement of RV for the diagnosis and follow-up of renal diseases, as well as for further studies, it is necessary to collect standard values in a standard population. The generation of reference parameters in the general population is essential for subsequent differentiation between health and disease states. Cohen et al., 2009 showed that renal volume could be used to stage CKD patients with progressing renal disease accurately [8]. To date, there have only been a few studies which provide reference values for RV in the general population [7,8,9,10], and it remains unclear what defines high and low RV. In this study, we used the MRI database of the Study of Health in Pomerania (SHIP) to gather reference values and possible determinants of RV.

In Germany, more than 2 million inhabitants suffer from CKD and more than 100,000 patients depend on renal replacement therapy [11,12]. The risk factors for the development and progression of CKD, such as diabetes and arterial hypertension, are well known and are an inherent part of the guidelines devised to prevent the progression and direct the therapy of CKD [13]. As shown by previous studies, changes in the glomerular filtration rate (GFR) are associated with changes in renal volume [14,15,16,17]. Therefore, in a second step of this study, we correlated selected risk factors of CKD as well as GFR with kidney volume within our study cohort. 

The main objective of our study is to describe reference values for renal volume in people from the general population without known kidney disease. 

## 2. Materials and Methods

### 2.1. Study Sample 

The rationale and design of the Study of Health in Pomerania (SHIP-TREND) have been presented in detail by Völzke et al., 2022 [18]. 

**Figure 1:**
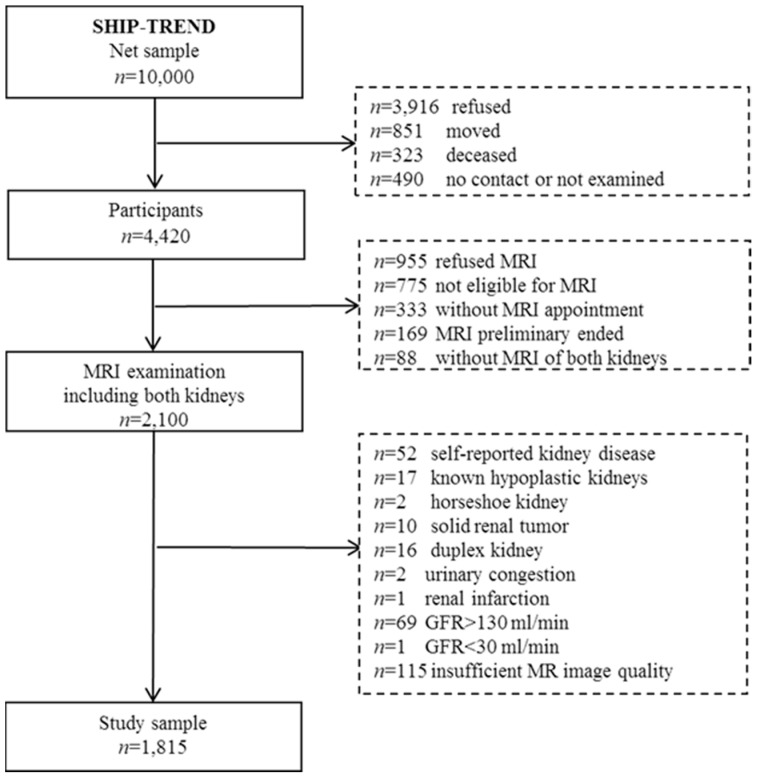
Flow chart of the study population from SHIPT-TREND.

SHIP-TREND is a population-based study. Participants were sampled from the general adult population, aged 20 to 79 years, in West Pomerania, Northeast Germany. We used a two-stage stratified cluster sample. Sample selection was supported by the residents’ registration offices of the Federal State of Mecklenburg/West Pomerania. A sample of 10,000 adults aged 20 to 79 years was drawn. Stratification variables were age, sex, and city/county of residence. The target sample size was chosen to obtain a final sample size similar to that of SHIP-START-0. From 2008 until 2011, 4420 men and women aged 20 to 79 years participated in SHIP-TREND. Among them, 2100 persons, aged 21 to 81 years, were eligible and willing to undergo whole-body MRI [18] (Figure 1). SHIP-TREND was one of the first studies worldwide to utilise MRI in a general population setting. 

The characteristics of MRI participants of eligible as well as non-eligible nonparticipants are described by Schmidt et al., 2016 [19]. MRI participants were better educated, more often employed, less likely to be smokers, and more likely to be married compared to eligible and non-eligible nonparticipants. MRI participants also had a higher mean SF-12 Physical Component Summary Score than the other two groups. There were a few differences between participants and nonparticipants in the SF-12 Mental Component Summary Score and the depression score.

In the presented cross-sectional study, we included these 2100 subjects who underwent whole-body MRI including both kidneys. We excluded participants with self-reported kidney disease (*n* = 52), known hypoplastic kidneys (*n* = 17), horseshoe kidney (*n* = 2), solid renal tumour (*n* = 10), duplex kidney (*n* = 16), urinary congestion (*n* = 2), renal infarction (*n* = 1), nephrectomy (*n* = 0), kidney stones (*n* = 0), and GFR > 130 mL/min (*n* = 69), as well as GFR < 30 mL/min (*n* = 1). Furthermore, participants with insufficient MR image quality were excluded (*n* = 115). The final analytical sample comprised 1815 participants (930 women) aged 21–81 years.

The study was approved by the Ethics Committee of the University of Greifswald (BB 39/08) and complies with the Declaration of Helsinki. All participants provided written informed consent.

### 2.2. MR Imaging and Renal Volumetry

Whole-body MR imaging was performed on a commercially available 1.5 T scanner (Magnetom Avanto, Siemens Medical Systems, Erlangen, Germany). All volunteers were examined in a supine position. Images were acquired using integrated coil elements and phased-array surface coils. Kidneys were visualised using coronar TIRM (Turbo-Inversion Recovery-Magnitude) sequences (slice thickness: 5 mm, gap: 1 mm, TR: 4891 msec, TE: 67 msec, flip angle: 180°). Imaging analysis and measurements were obtained with the Picture Archiving and Communication System IMPAX (version: 6.5.2.114, Agfa HealthCare, Mortsel, Belgium). Renal volumetry was performed with the IMPAX Volume Viewing 3D tool (version 3.0, Agfa HealthCare, Mortsel, Belgium).

Volumetry was carried out independently by three readers (SP, FF, WJ) blinded to the demographic data. All readers had previously undergone training under the supervision of a board-certified radiologist with more than eleven years’ experience in abdominal MRI. To test the intra- and interobserver variability, all readers performed twofold renal volumetry on twenty datasets not included in this series. The Bland–Altman analysis revealed a mean bias > ±4% with limits of agreement < ±12%.

Before starting renal volumetry, each reader separately rated the image quality of both kidneys as sufficient (complete depiction of both kidneys with clear delineation of renal borders from perirenal fat) or insufficient (incomplete depiction of at least one kidney, occurrence of artefacts obscuring the renal boundaries). 

Renal volumetry was performed for each kidney separately and semi-automatically. Volumetry was started by outlining the renal boundaries in selected images. The volumetry tool generated the missing regions of interest automatically until the whole kidney was covered. After correcting eventually occurring inaccuracies manually, the tool generated the renal volume automatically. The volumetry included the renal cortex and medulla, defined as RV, whereas renal cysts, the renal pelvis, and renal vessels were excluded. 

### 2.3. Clinical and Laboratory Measurements

The investigations were conducted through computer-assisted personal interviews and self-administered questionnaires, physical and instrumental examinations, and laboratory analyses, and were carried out by trained and certified experts in a standardised manner at the University of Medicine, Greifswald. Non-fasting blood samples were taken between 7:00 a.m. and 4:00 p.m. and analysed in the central laboratory of the University of Medicine, Greifswald. As part of the quality assurance, all analytical laboratories were subjected to semi-annual official national German tests and duplicates of the laboratory blood samples were examined every week within the framework of internal quality assurance [18]. 

Serum creatinine concentrations were measured enzymatically (Dimension VISTA, Siemens Healthcare Diagnostics, Eschborn, Germany), while urinary creatinine and albumin concentrations were measured with a nephelometric assay (BN ProSpec Analyzer, Dade Behring, Deerfield, IL, USA). We used the CKD epidemiology collaboration (CKD-EPI) equations to estimate GFR [20]:

If male: 141 × min (Scr/0.9, 1) − 0.411 × max (Scr/0.9, 1) − 1.209;

If female: 141 × min (Scr/0.7, 1) − 0.329 × max (Scr/0.7, 1) − 1.209.

SCr means serum creatinine, and Q-values are mean values of serum creatinine for age-/sex-specific healthy populations.

### 2.4. Statistical Analysis

Characteristics in women and men were summarised by number and percentage or median and interquartile range (see Table 1). Differences between women and men were evaluated by χ^2^-test or Wilcoxon rank-sum (Mann–Whitney) test.

Right and left renal volumes with and without body surface area (BSA) indexation were compared among three age groups (22–39 years, 40–59 years, 60–81 years) by median and interquartile range and tested by a Kruskal–Wallis test separately in women and men. For lower reference values, 5th percentiles were chosen and presented for each age group. Reference intervals of renal volume according to continuous age and glomerular filtration rate were displayed graphically by using generalised least squares fractional polynomial regression models (Stata module: XRIGLS). The 5th and 95th percentiles were chosen as the lower and upper limits of a reference range (see Table 2). 

Associations between risk factors and right and left renal volume were analysed by multivariable linear regression, and β-coefficients with 95% confidence intervals were provided. Risk factors for renal volume included age, sex, smoking status, BSA, systolic and diastolic blood pressure, type 2 diabetes, HDL-C, LDL-C, glomerular filtration rate, and uric acid. 

The normality and homoscedasticity of regression residuals were checked graphically and multicollinearity was excluded by calculating variance inflation factors. A value of *p* < 0.05 was considered statistically significant. Statistical analyses were performed using Stata 14.1 (Stata Corporation, College Station, TX, USA).

## 3. Results

Baseline characteristics of women (*n* = 930) and men (*n* = 885) are provided in Table 1. The ages of women (median = 52 years) and men (median = 51) were similar. Men had a lower proportion of never-smokers (31%) and a higher proportion of hypertension (50%) compared to women (never-smoker: 47%; hypertension: 37%). The estimated glomerular filtration rate (eGFR), uric acid, and right and left renal volume were higher in men than in women (all *p* < 0.001). 

**Table 1:** Baseline and kidney characteristics of the study sample (SHIP-TREND; *n* = 1815).

	Women	Men	
Parameter	*n* = 930	*n* = 885	*p*-Value *
Age (years)	52 (41; 62)	51 (41; 62)	0.718
Smoking status			<0.001
Never-smoker	437 (47.0%)	270 (30.6%)	
Ex-smoker	280 (30.1%)	398 (45.1%)	
Current smoker	212 (22.8%)	214 (24.3%)	
Body mass index (kg/m^2^)	26.5 (23.3; 30.5)	27.8 (25.5; 30.5)	<0.001
Body surface area (m^2^)	1.78 (1.68; 1.89)	2.05 (1.94; 2.15)	<0.001
Systolic BP (mmHg)	119 (109; 130)	132 (123; 142)	<0.001
Diastolic BP (mmHg)	74 (69; 80)	80 (73; 86)	<0.001
Hypertension	345 (37.1%)	439 (49.9%)	<0.001
Diabetes mellitus (Typ-2)	58 (6.2%)	73 (8.3%)	0.994
HbA1c (%)	5.2 (4.8; 5.6)	5.3 (4.9; 5.6)	<0.001
HDL-C (mmol/L)	1.58 (1.35; 1.85)	1.25 (1.07; 1.47)	<0.001
LDL-C (mmol/L)	3.33 (2.76; 4)	3.43 (2.77; 4)	0.697
eGFR (mL/min per 1.73 m^2^)	84.9 (73.4; 98.75)	88.6 (77.8; 100.4)	<0.001
Uric acid (mmol/L)	238 (198; 278)	319 (278; 364)	<0.001
Albumin i.U. (mg/L) ^#^	7.9 (5.6; 13.2)	9 (6.1; 15.2)	0.003
Urinary albumin/creatinin ratio (uACR, g/g) ^##^	8.9 (5.81; 16.35)	5.87 (4.21; 11.08)	<0.001
Left parenchyma volume (mL)	144 (125; 165)	180 (160; 203)	<0.001

Data are given as number (percentage) or median (25th and 75th percentile). BP, blood pressure; HbA1c, hemoglobin A1c; HDL-C, high density lipoprotein cholesterin; LDL-C, low density lipoprotein cholesterin. * *p*-values are from χ^2^ test or Wilcoxon rank-sum (Mann–Whitney) test. ^#^ *n* = 1272. ^##^ *n* = 1270.

**Figure 2:**
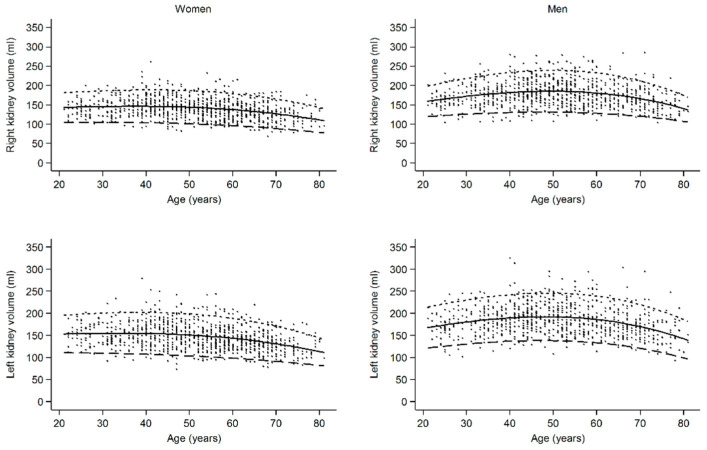
Continuous reference values and normal ranges for renal volume according to age, showing a decreasing trend in women that is stronger in older ages and an upside-down U-shaped relation in men. Dots, observations; solid line, median; large dashed line, 5th percentile; small dashed line; 95th percentile.

The female lower reference value of right renal volume decreased from 106 mL in the youngest age group to 92 mL in the oldest age group and that of the left renal volume from 114 mL to 93 mL (*p* < 0.001) (Table 2). In men, the highest reference values of renal volume could be observed in the middle age group (right: 134 mL; left: 142 mL), whereas reference values did not differ between youngest (right: 123 mL; left: 129 mL) and oldest age group (right: 123 mL; left: 128). Continuous reference values and normal ranges for renal volume according to age are displayed in Figure 2, with a clear decreasing trend in women that is stronger in older ages and an upside-down U-shape relation in men. The same differences in renal volume between men and women and among age groups were seen after the BSA indexation of renal volume (Table 2).

**Table 2:** Median renal parenchyma volume with lower reference values (5th percentile) in women and men according to different age groups (*n* = 1815).

		Women		Men	
	Age (Years)	Median (25th, 75th)	Reference Value (5th Percentile)	Median (25th, 75th)	Reference Value (5th Percentile)
Right kidney volume		138 (121; 156)	99	174 (154; 197)	126
	22–39	141 (126; 160)	106	172 (153; 192)	123
	40–59	142 (126; 161)	102	180 (162; 206)	134
	60–81	129 (111; 143)	92	164 (149; 184)	123
	*p*-value	<0.001		<0.001	
Left kidney volume		144 (125; 165)	102	180 (160; 203)	132
	22–39	150 (134; 169)	114	179 (161; 200)	129
	40–59	149 (128; 170)	109	190 (168; 215)	142
	60–81	131 (115; 148)	93	168 (150; 186)	128
	*p*-value	<0.001		<0.001	
Right kidney volume/BSA		77.4 (68.8; 86.4)	57.3	84.6 (77.0; 93.9)	65.4
	22–39	79.6 (72.0; 88.9)	60.0	84.0 (76.3; 89.9)	64.5
	40–59	79.7 (71.6; 88.0)	60.2	89.2 (79.3; 98.4)	67.6
	60–81	71.1 (63.9; 80.1)	53.7	80.4 (74.7; 88.8)	63.8
	*p*-value	<0.001		<0.001	
Left kidney volume/BSA		80.2 (71.6; 90.3)	59.6	87.7 (78.9; 97.6)	67.7
	22–39	84.0 (77.3; 92.6)	63.7	87.2 (78.9; 95.9)	68.4
	40–59	83.1 (74.2; 93.2)	63.0	92.0 (82.3; 101.8)	70.8
	60–81	73.3 (65.0; 81.8)	55.2	82.0 (75.4; 89.8)	64.4
	*p*-value	<0.001		<0.001	

BSA, body surface area. *p*-values are from Kruskal–Wallis test for differences in kidney parameters among age groups.

**Figure 3:**
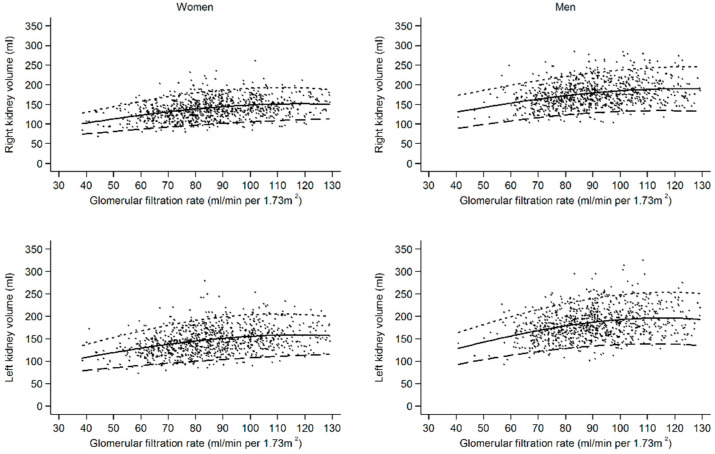
Association between estimated glomerular filtration rate (eGFR) and renal volume (RV). The same eGFR is associated with a higher renal volume in men compared to women. Dots, observations; solid line, median; large dashed line, 5th percentile; small dashed line; 95th percentile.

In multivariable linear regression models, current smoking (β = 14.96, 95% CI 12.12; 17.79), BSA (β = 97.66, 95% CI 90.4; 104.93), diastolic blood pressure (β = 0.17, 95% CI 0.01; 0.32), and eGFR (β = 0.57, 95% CI 0.50; 0.65) were positively associated with both left and right renal volume, whereas uric acid (β = −0.03, 95% CI −0.05; −0.01) demonstrated an inverse association with renal volume (Table 3). HDL-C (β = −4.8, 95% CI −8.1; −1.5) and type 2 diabetes were only associated with right renal volume. The positive association between eGFR and renal volume is also displayed in Figure 3. Dichotomised increased eGFR > 120 mL/min/1.73 m^2^ (*n* = 68) is also independently associated with higher right kidney volume (β = 14.09; 95% CI 8.24; 19.95; *p* < 0.001) and left kidney volume (β = 11.61; 95% CI 5.32; 17.90; *p* < 0.001). Interestingly, the same eGFR shows a higher renal volume in men compared to women (Figure 3).

Albumin in urine [mg/L] and urinary albumin-to-creatinine ratio (uACR) [g/g] were within the normal range and did not correlate with kidney volume, either in men or in women (Table 3).

As a sensitivity analysis corroborating our reported results for the total cohort, we divided our cohort into 248 healthy subjects (none of the following conditions: hypertension, diabetes, current smoking, myocardial infarction, angina pectoris, stroke, atrial fibrillation, or heart failure; LDL-cholesterol < 3.0 mmol/L) and remaining unhealthy 1553 individuals. In those 1553 subjects with one or more risk factor or comorbidity, there were positive associations between kidney volume and male sex (*p* < 0.001), current smoking (*p* < 0.001), body surface area (*p* < 0.001), diastolic blood pressure (only left kidney volume) (*p* = 0.037), low HDL (only right kidney volume) (*p* = 0.003), diabetes mellitus (only right kidney volume) (*p* = 0.017), eGFR (*p* < 0.001), and a negative association with uric acid (*p* = 0.002). In the 248 healthy subjects, there were positive associations between kidney volume and body surface area (*p* < 0.001), eGFR (*p* < 0.001), and a negative association for uric acid (only left kidney) (*p* = 0.05) (Supplemental Tables S1–S6).

## 4. Discussion

### Summary of the Main Results

In the chosen reference population, the following facts have been found and documented:

(1) Reference values for renal volumes are different for age groups and sex.
(2) For any given age and even when indexed to BSA, female kidneys are smaller than male kidneys.
(3) Renal volume is associated positively with eGFR, but for any chosen eGFR, renal volume in females is lower compared to males.
(4) Left kidneys are larger than right kidneys in both males and females.
(5) Hypertension, diabetes, and smoking were associated with larger renal volumes.
(6) Overt cardiovascular risk factor accumulation in middle-aged males serves as a potential explanation for hyperfiltration and renal hypertrophy in this group.

All kidney diseases show a change in renal volume during the course of the disease, with the majority of CKD ultimately resulting in a loss of volume [21,22]. However, renal volume may also be higher in early stages of a disease or in the presence of certain risk factors—presumably representing glomerular hypertrophy and a hyperfiltration state [23]. This fact is best known for diabetic kidney disease, but may also occur in arterial hypertension or smoking [10,24].

Previous studies showed that renal function, determined by eGFR, is clearly associated with renal volume, which makes renal volume an important parameter for the diagnosis and follow-up of renal diseases [10,14,15,16,17]. In clinical practice, renal dimensions are mostly assessed by US due to the good visualisation of the kidneys, widespread use, and low cost. A disadvantage of US is that kidney volume can only be estimated from unidimensional measurements. The use of cross-sectional imaging such as CT and MRI enables the true volumetric assessment of the kidneys. MRI is a highly reliable and valid tool for calculating the RV [6,7].

Renal volume is influenced by several physiological and pathological factors. First, the average RV of the left kidney is greater compared to the right kidney in both men and women [7,8,9,10]. Even though the difference is only a few millilitres, this was also seen in our study across all age groups and in both sexes. A possible explanation is the high volume of the liver, which limits the volume of the right kidney, compared to the left.

In our reference population, the median renal volume in men for the right kidney was 174 mL and 180 mL for the left kidney; in women, RV was 138 mL for the right kidney and 144 mL for the left kidney. Even when indexed to BSA, renal volume in male kidneys remained larger than in female kidneys. This was also reported in studies with smaller cohorts [8,9,15,16,25,26], as well as in the Framingham Heart Study [10]. Only Van den Dool et al., 2005 could not detect significant gender differences, probably attributable to a small cohort size [5]. Cohen et al., 2009 showed similar results for the RV of right and left male kidneys, whereas female RV was reported to be slightly higher than in our study [8].

In women, renal volume was nearly constant until the age of 59 years and decreased in the age group of 60–81 years. As shown in the continuous graph in Figure 2, volume loss began after the age of 50 years. This corresponds with the findings of other groups, describing a similar trend of decreasing total renal volume (TRV) [9,27] or cortical volume [5,16] in both older men and women. Additionally, our data show that RV in women is constant until the age of 50 years. In men, we found the highest RV in the middle-aged group (40–59 years) and lower volumes in the younger (22–39 years) and in the oldest age group (60–81 years). This was also reported by Roseman et al., 2017 [10]. This could be interpreted alongside the glomerular hyperfiltration/hypertrophy theory: in a high risk population, patients in the third tertile of kidney length (11.7–16.1 cm) were at higher risk of cardiovascular mortality and cardiovascular events, while patients in the first tertile of kidney length (7.8–10.8 cm) were not [24].

As shown in Figure 3 and Table 3, there was a significant positive association between RV and eGFR (*p* < 0.001). This effect was slightly stronger in males than in females. The correlation between eGFR and RV is confirmed by most studies [10,15,16,25,28]. As Johnson et al., 2011 reported within their study cohort, mGFR and renal parenchymal volume correlated even more strongly than measured GFR (mGFR) and creatinine-based equations [15]. As mentioned before, eGFR has often been reported as a suitable parameter for assessing renal function [14,15,16,17]. Due to the strong correlation between RV and GFR, which is supported by our study, RV can also aid in the diagnosis and follow-up of renal diseases.

Within our study population, we also found a clear positive correlation between RV and body surface area (BSA), similar to the results of Johnson et al., 2011 [15]. In contrast, Cheong et al., 2007 did not see any correlation between these two parameters in women, but a modest correlation in male subjects [9]. Roseman et al., 2017 revealed BSA as the highest risk factor for high TRV [10].

In CKD, high blood pressure is commonly found. Within our reference population, we could only show a significant association between RV and diastolic blood pressure (*p* < 0.05), whereas we detected no correlation between systolic blood pressure and RV. This is consistent with results from previous studies that also reported no significant association between blood pressure and RV [10,16,26]. The increasing proportion of participants with well-controlled hypertension in the SHIP-study cohorts over time may explain the lack of association with high blood pressure [29]. Nevertheless, Roseman et al., 2017 [10] found no association even when extending the definition of hypertension.

There was no significant association between type 2 diabetes and left renal volume within our study cohort, but a slight association with increasing right renal volume. In contrast, Roseman et al., 2017 [10] found a higher prevalence of diabetes in high renal volume compared to normal or lower renal volume. Smaller study groups, however, described a correlation between diabetes and RV [15,25,26,30]. The inconsistencies may result from limited numbers of diabetic subjects in population-based MRI studies.

In our study population, a correlation between smoking status and RV could be found. Current smokers had a significant higher renal volume in both kidneys (*p* < 0.001) in comparison to non-smokers and ex-smokers. The same was observed for total renal volume by other groups [10,16,24,25]. Different study groups found an impaired kidney function, defined as proteinuria and hyperfiltration (eGFR > 130 mL/min), in current smokers but not in ex-smokers [16,31,32].

Previous studies suggested that uric acid could be a predictor for the progression of CKD [33,34]. This could be supported by our study, where in the multivariable analysis a low renal volume was associated with increasing levels of uric acid.

We can also confirm the findings of Wang et al., 2014, who found an inverse correlation between kidney volume in CT scans and HDL-cholesterol, and who took this as evidence for HDL as a protective factor in cortical changes in early CKD [16], again interpreting a high RV as an expression of glomerular hypertrophy and stress.

Non-traditional cardiovascular risk factors such as anaemia, C-reactive protein, or frailty markers were of very low prevalence in our general population sample. Additionally, non-traditional risk factors so far are not known to associate with renal volume.

As a limitation of our study, we report a possible selection bias as we see differences in included versus excluded subjects in MRI SHIP-TREND study participants [19]. Another limitation is the lack of a comorbidity scale such as the Charlson Comorbidity Scale in SHIP-TREND. Single comorbidities are shown in Table 1 and Table 4.

We observed clear associations of RV with age, sex, eGFR, BSA, smoking status, and level of HDL-C, as well as uric acid. According to the hyperfiltration theory, cardiovascular risk factor accumulation in middle-aged males may explain the larger RV in this group. The analysis of single renal volumes could confirm most of the results of studies using the total renal volume. To the best of our knowledge, the presented reference values and results are based on one of the largest studies for renal volumetry with the broadest age range so far. In future, these reference parameters could be used for comparisons with the renal volume of diseased kidneys. However, to establish MRI-based renal volumetry as a tool in daily clinical decision making, further studies on areas such as longitudinal changes in renal volume during CKD progression are needed.

## Figures and Tables

**Table 3:** Association between risk factors and kidney volume (*n* = 1805).

	Right Kidney Volume		Left Kidney Volume	
Risk Factor	β (95% CI)	*p*-Value	β (95% CI)	*p*-Value
Men	8.97 (5.91; 12.03)	<0.001	9.43 (6.1; 12.75)	<0.001
Age	0.10 (0.00; 0.19)	0.049	0.00 (−0.11; 0.10)	0.948
Smoking status				
Never smoker	Ref		Ref	
Ex-smoker	2.31 (−0.12; 4.75)	0.063	1.93 (−0.72; 4.59)	0.153
Current smoker	14.96 (12.12; 17.79)	<0.001	16.03 (12.94; 19.11)	<0.001
Body surface area	97.66 (90.4; 104.93)	<0.001	100.55 (92.64; 108.47)	<0.001
Systolic BP	0.00 (−0.10; 0.10)	0.996	−0.02 (−0.13; 0.08)	0.648
Diastolic BP	0.17 (0.01; 0.32)	0.032	0.22 (0.05; 0.39)	0.011
Diabetes mellitus (Typ-2)	5.60 (1.31; 9.90)	0.011	3.99 (−0.69; 8.67)	0.095
HDL-C	−4.55 (−7.88; −1.23)	0.007	−3.22 (−6.84; 0.40)	0.081
LDL-C	0.35 (−0.82; 1.51)	0.559	0.31 (−0.95; 1.58)	0.629
Glomerular filtration rate	0.57 (0.5; 0.65)	<0.001	0.54 (0.46; 0.62)	<0.001
Uric acid	−0.03 (−0.05; −0.01)	0.001	−0.04 (−0.06; −0.02)	<0.001

BP, blood pressure; HDL-C, high density lipoprotein cholesterin; LDL-C, low density lipoprotein cholesterin. β-coefficients are from linear regression.

**Table 4:** Characteristics of the study sample: cardiovascular risk factor differences among age groups (SHIP-TREND; *n* = 1815).

	Age Groups (Years)			
	22–39	40–59	60–81	
Parameter	*n* = 384	*n* = 888	*n* = 543	*p*-Value *
Men	199 (51.8%)	420 (47.3%)	266 (49%)	0.331
Smoking status				<0.001
Never-smoker	140 (36.7%)	312 (35.2%)	255 (47.1%)	
Ex-smoker	101 (26.4%)	333 (37.5%)	244 (45%)	
Current smoker	141 (36.9%)	242 (27.3%)	43 (7.9%)	
Body mass index (kg/m^2^)	24.9 (22.7; 28.1)	27.3 (24.4; 30.4)	28.8 (26.3; 31.8)	<0.001
Body surface area (m^2^)	1.92 (1.78; 2.06)	1.92 (1.76; 2.07)	1.91 (1.76; 2.06)	0.617
Systolic BP (mmHg)	119 (109; 131)	125 (113; 137)	132 (123; 144)	<0.001
Diastolic BP (mmHg)	74 (68; 80)	79 (72; 85)	77 (71; 83)	<0.001
Hypertension	53 (13.9%)	357 (40.3%)	374 (69%)	<0.001
Diabetes mellitus (Typ-2)	4 (1%)	38 (4.3%)	89 (16.4%)	<0.001
HbA1c (%)	5.0 (4.7; 5.3)	5.2 (4.9; 5.5)	5.5 (5.2; 5.8)	<0.001
HDL-C (mmol/L)	1.41 (1.17; 1.66)	1.42 (1.2; 1.7)	1.40 (1.17; 1.69)	0.201
LDL-C (mmol/L)	2.96 (2.38; 3.54)	3.52 (2.94; 4.07)	3.45 (2.84; 4.11)	<0.001
eGFR (mL/min per 1.73 m^2^)	95.2 (86.45; 107.8)	88.4 (78.5; 100.5)	76.3 (67.3; 87)	<0.001
Uric acid (mmol/L)	263 (216.5; 308)	275 (222; 328)	294.5 (247; 352)	<0.001
Albumin i.U. (mg/L)	8.1 (6; 13)	8.1 (5.6; 12.7)	9.8 (6.3; 20.1)	<0.001
Right parenchyma volume (mL)	156 (138; 179)	160 (137; 185)	146 (124; 168)	<0.001
Left parenchyma volume (mL)	166 (144; 186)	167 (143; 194)	148 (128; 172)	<0.001

Data are given as a number (percentage) or median (25th and 75th percentile). * *p*-values are from χ^2^ test or Kruskal–Wallis test. HbA1c, hemoglobin A1c; HDL-C, high density lipoprotein cholesterin;

## Data Availability

Data from the “Study of Health of Pomerania” are available from the University of Medicine, Greifswald, Germany, but restrictions apply to the availability of these data, which were used under license for the current study, and so are not publicly available. Data are, however, available upon reasonable request at https://transfer.ship-med.uni-greifswald.de/FAIRequest/ and with permission of the University of Medicine, Greifswald.

## Supplementary Materials

The following supporting information can be downloaded at: https://www.mdpi.com/article/10.3390/jcm13030769/s1, Table S1. Median renal parenchyma volume with lower reference values (5th percentile) in women and men according to CKD stages G1-3 (*n* = 1813). In our general population sample, there were only 2 persons in CKD G4-5. Table S2. Association between risk factors and kidney volume including urinary albumin-to-creatinine ratio (uACR) (*n* = 1260). Table S3. Characteristics of the study sample according to CKD stages G1-3 (SHIP-TREND; *n* = 1813). In our general population sample, there were only 2 persons with CKD G4-5. Table S4. Association between risk factors and kidney volume in women (n = 930) and men (*n* = 885). Table S5. Association between risk factors and kidney volume in healthy subjects (*n* = 248). Table S6. Association between risk factors and kidney volume in unhealthy subjects (*n* = 1553).
