# Supplementary material for: Age- and Sex-Specific Reference Values for Renal Volume and Association with Risk Factors for Chronic Kidney Disease in a General Population—An MRI-Based Study
Source: J Clin Med. 2024 Jan 29;13(3):769. doi: 10.3390/jcm13030769 (PMC10856696; doi:10.3390/jcm13030769)
Supplement: Supplementary file 1 [file jcm-13-00769-s001.zip › jcm-2767448-supplementary.pdf]

Supplemental Table S1. Median renal parenchyma volume with lower reference values (5th percentile) in women and men according to CKD stages G1-3 (N=1813). In our general population sample, there were only 2 persons in CKD G4-5.

		Women		Men	
	eGFR (ml/min per 1.73m²)	Median (25th, 75th)	Reference value (5th percentile)	Median (25th, 75th)	Reference value (5th percentile)
Right kidney volume		138 (121;157)	99	174 (154;197)	126
	≥90	146 (131;164)	105	181 (162;208)	142
	60 – 89	134 (118;152)	99	166 (148;188)	124
	30 – 59	117 (100;131)	85	168 (118;194)	110
	p-value	<0.001		<0.001	
Left kidney volume		144 (125;165)	102	180 (160;203)	132
	≥90	151 (135;172)	114	190 (167;215)	143
	60 – 89	141 (122;162)	101	173 (154;195)	129
	30 – 59	122 (110;138)	82	152 (112;187)	93
	p-value	<0.001		<0.001	
Right kidney volume/BSA		77.4 (68.8;86.4)	57.3	84.6 (77.0;93.9)	65.4
	≥90	83.0 (74.0;90.8)	62.9	89.0 (81.7;99.1)	72.3
	60 – 89	75.5 (67.4;83.3)	56.9	80.4 (73.7;90.1)	64.2
	30 – 59	63.3 (56.5;70.3)	45.5	79.6 (61.7;88.6)	57.2
	p-value	<0.001		<0.001	
Left kidney volume/BSA		80.2 (71.6;90.3)	59.6	87.7 (78.9;97.6)	67.7
	≥90	85.5 (76.5;95.6)	65.4	92.7 (83.3;103.3)	73.2
	60 – 89	78.4 (70.4;88.4)	59.4	84.4 (76.6;92.3)	67.1
	30 – 59	67.4 (59.1;73.3)	47.1	74.8 (55.7;88.6)	53.9
	p-value	<0.001		<0.001	

p-values are from Kruskal Wallis test for differences of kidney parameters among age groups

Supplementary Table S2. Association between risk factors and kidney volume including urinary albumin-to-creatinine ratio (uACR) **(n=1260).**

Risk factor	Right kidney volume β (95%CI)	p-value	Left kidney volume β (95%CI)	p-value
Men	8.95 (5.11; 12.79)	<0.001	9.90 (5.71; 14.08)	<0.001
Age	0.01 (-0.10; 0.13)	0.842	-0.15 (-0.28; -0.03)	0.017
Smoking status				
Never smoker	Ref		Ref	
Ex-smoker	2.71 (-0.33; 5.75)	0.081	3.39 (0.08; 6.71)	0.045
Current smoker	13.9 (10.4; 17.5)	<0.001	15.0 (11.1; 18.8)	<0.001
Body surface area	97.7 (88.6; 106.7)	<0.001	99.6 (89.7; 109.4)	<0.001
Systolic BP	0.03 (-0.08; 0.15)	0.588	0.02 (-0.10; 0.15)	0.708
Diastolic BP	0.12 (-0.07; 0.31)	0.227	0.16 (-0.05; 0.37)	0.125
Diabetes mellitus (Typ-2)	4.35 (-0.58; 9.28)	0.084	2.77 (-2.61; 8.15)	0.313
HDL-C	-5.53 (-9.7; -1.35)	0.009	-3.26 (-7.8; 1.29)	0.161
LDL-C	0.11 (-1.32; 1.54)	0.878	-0.23 (-1.78; 1.33)	0.773
Glomerular filtration rate	0.56 (0.47; 0.65)	<0.001	0.48 (0.39; 0.58)	<0.001
Uric acid	-0.03 (-0.05; -0.01)	0.01	-0.04 (-0.06; -0.01)	0.002
Urinary albumin-to-creatinine-ratio (uACR)	0.01 (0.00; 0.02)	0.206	0.00 (-0.01; 0.01)	0.937

β-coefficients are from linear regression

Supplemental Table S3. Characteristics of the study sample according to CKD stages G1-3 (SHIP-TREND; n=1,813). In our general population sample, there were only 2 persons with CKD G4-5.

	eGFR (ml/min per 1.73m²)			
	CKD G1	CKD G2	CKD G3	
Parameter	n=754	n=990	n=69	p-value*
Women	348 (46.1%)	526 (53.1%)	54 (78.3%)	0.001
Age (years)	45 (36; 55)	55 (45; 65)	69 (64; 74)	<0.001
Smoking status				<0.001
Never-smoker	249 (33.2%)	425 (43%)	32 (46.4%)	
Ex-smoker	274 (36.5%)	372 (37.6%)	31 (44.9%)	
Current smoker	228 (30.4%)	192 (19.4%)	6 (8.7%)	
Body mass index (kg/m²)	26.5 (23.7; 29.9)	27.8 (24.8; 30.8)	30.1 (26.6; 33.6)	<0.001
Body surface area (m²)	1.93 (1.77; 2.06)	1.91 (1.76; 2.07)	1.87 (1.74; 2.05)	0.367
Systolic BP (mmHg)	125 (113; 137)	127 (116; 138)	134 (124; 147)	<0.001
Diastolic BP (mmHg)	77 (71; 84)	77 (71; 84)	78 (72; 82)	0.991
Hypertension	260 (34.6%)	467 (47.3%)	56 (81.2%)	<0.001
Diabetes mellitus (Typ-2)	27 (3.6%)	82 (8.3%)	22 (31.9%)	<0.001
HbA1c (%)	5.2 (4.8; 5.5)	5.3 (4.9; 5.6)	5.7 (5.4; 6)	<0.001
HDL-C (mmol/l)	1.43 (1.20; 1.70)	1.4 (1.17; 1.69)	1.36 (1.09; 1.73)	0.236
LDL-C (mmol/l)	3.31 (2.68; 3.96)	3.43 (2.86; 4.03)	3.38 (2.61; 4.21)	0.047
Uric acid (mmol/l)	270 (219; 319)	280 (233; 336)	347 (299; 406)	<0.001
Albumin i.U. (mg/l)	8.0 (5.7; 13)	8.9 (6; 15.2)	10 (5.7; 19.3)	0.010
Right Parenchyma volume (ml)	165 (143; 187)	149 (128; 172)	119 (107; 140)	<0.001
Left Parenchyma volume (ml)	170 (148; 198)	156 (134; 180)	126 (112; 141)	<0.001

Data are given as number (percentage) or median (25th and 75th percentile). *p-values are from χ^2 test or Kruskal Wallis test

CKD G1: eGFR≥90 ml/min/1.73 m², CKD G2: eGFR 60-89 ml/min/1.73 m²; CKD G3: eGFR 30-59 ml/min/1.73 m²

Supplemental Table S4. Association between risk factors and kidney volume in women (n=930) and men (n=885).

	Right kidney volume β (95%CI)	p-value	Left kidney volume β (95%CI)	p-value
**WOMEN**				
Age	-0.06 (-0.2; 0.07)	0.358	-0.16 (-0.31; 0)	0.044
Smoking status				
Never smoker	Ref		Ref	
Ex-smoker	0.44 (-2.65; 3.52)	0.781	-0.51 (-4; 2.97)	0.773
Current smoker	11.8 (8.3; 15.4)	<0.001	14.0 (9.98; 18.0)	<0.001
Body surface area	81.8 (72.6; 90.9)	<0.001	87.7 (77.3; 98.1)	<0.001
Systolic BP	-0.02 (-0.15; 0.1)	0.705	-0.03 (-0.17; 0.11)	0.666
Diastolic BP	0.16 (-0.05; 0.38)	0.103	0.17 (-0.07; 0.41)	0.166
Diabetes mellitus (Typ-2)	3.07 (-2.77; 8.92)	0.303	3.34 (-3.25; 9.94)	0.32
HDL-C	-6.4 (-10.5; -2.35)	0.002	-5.38 (-9.95; -0.8)	0.021
LDL-C	-0.09 (-1.61; 1.43)	0.911	-0.26 (-1.98; 1.45)	0.762
eGFR	0.49 (0.4; 0.58)	<0.001	0.43 (0.33; 0.53)	<0.001
Uric acid	-0.03 (-0.06; -0.01)	0.013	-0.06 (-0.08; -0.03)	<0.001
**MEN**				
Age	0.25 (0.11; 0.39)	0.001	0.15 (0; 0.29)	0.054
Smoking status				
Never smoker	Ref		Ref	
Ex-smoker	3.08 (-0.79; 6.96)	0.119	3 (-1.12; 7.11)	0.154
Current smoker	17.0 (12.5; 21.5)	<0.001	16.8 (12.0; 21.5)	<0.001
Body surface area	114.7 (103.4; 125.9)	<0.001	114.8 (102.8; 126.7)	<0.001
Systolic BP	0.08 (-0.07; 0.22)	0.313	0.05 (-0.11; 0.21)	0.558
Diastolic BP	0.07 (-0.15; 0.3)	0.534	0.15 (-0.09; 0.39)	0.222
Diabetes mellitus (Typ-2)	7.19 (0.97; 13.42)	0.023	4.24 (-2.37; 10.85)	0.208
HDL-C	-3.68 (-9.06; 1.71)	0.181	-1.97 (-7.69; 3.76)	0.500
LDL-C	1.54 (-0.25; 3.33)	0.091	1.61 (-0.29; 3.51)	0.097
eGFR	0.67 (0.56; 0.79)	<0.001	0.66 (0.54; 0.79)	<0.001
Uric acid	-0.02 (-0.05; 0)	0.102	-0.03 (-0.05; 0)	0.051

β-coefficients are from multivariable linear regression models.

Supplemental Table S5. Association between risk factors and kidney volume in healthy subjects (n=248)

Risk factor	Right kidney volume β (95%CI)	p-value	Left kidney volume β (95%CI)	p-value
Men	5.92 (-3.08; 14.91)	0.196	9.46 (-0.34; 19.3)	0.058
Age	0.12 (-0.11; 0.35)	0.303	-0.03 (-0.28; 0.22)	0.804
Smoking status				
Never smoker	Ref		Ref	
Ex-smoker	-0.01 (-5.43; 5.42)	0.999	-0.40 (-6.32; 5.51)	0.893
Current smoker	-		-	
Body surface area	100.8 (81.4; 120.2)	<0.001	100.1 (79.0; 121.3)	<0.001
Systolic BP	-0.09 (-0.46; 0.28)	0.637	-0.03 (-0.44; 0.37)	0.877
Diastolic BP	0.32 (-0.20; 0.84)	0.223	0.36 (-0.20; 0.92)	0.206
Diabetes mellitus (Typ-2)	-			
HDL-C	-0.03 (-8.63; 8.58)	0.995	-3.00 (-12.4; 6.37)	0.529
LDL-C	-6.00 (-13.1; 1.08)	0.096	-3.30 (-11.0; 4.41)	0.400
Glomerular filtration rate	0.45 (0.27; 0.63)	<0.001	0.48 (0.28; 0.67)	<0.001
Uric acid	-0.04 (-0.10; 0.02)	0.166	-0.09 (-0.16; -0.03)	0.005
Albumin/Creatinin-Ratio	0.00 (-0.05; 0.05)	0.900	0.00 (-0.06; 0.06)	0.993

β-coefficients are from linear regression

Supplemental Table S6. Association between risk factors and kidney volume in unhealthy subjects (n=1,553)

Risk factor	Right kidney volume β (95%CI)	p-value	Left kidney volume β (95%CI)	p-value
Men	9.77 (6.45; 13.1)	<0.001	9.52 (5.9; 13.13)	<0.001
Age	0.09 (-0.02; 0.20)	0.097	0.01 (-0.11; 0.13)	0.896
Smoking status				
Never smoker	Ref		Ref	
Ex-smoker	2.62 (-0.11; 5.35)	0.060	2.42 (-0.56; 5.39)	0.111
Current smoker	15.0 (11.8; 18.1)	<0.001	16.6 (13.2; 20.0)	<0.001
Body surface area	98.0 (90.1; 106.0)	<0.001	101.5 (92.8; 110.2)	<0.001
Systolic BP	0.01 (-0.09; 0.11)	0.888	-0.02 (-0.13; 0.09)	0.705
Diastolic BP	0.13 (-0.03; 0.30)	0.122	0.19 (0.01; 0.37)	0.037
Diabetes mellitus (Typ-2)	5.33 (0.96; 9.71)	0.017	3.76 (-1.01; 8.54)	0.122
HDL-C	-5.44 (-9.07; -1.80)	0.003	-3.34 (-7.31; 0.63)	0.099
LDL-C	0.67 (-0.62; 1.96)	0.311	0.56 (-0.85; 1.97)	0.437
Glomerular filtration rate	0.59 (0.51; 0.67)	<0.001	0.55 (0.46; 0.63)	<0.001
Uric acid	-0.03 (-0.05; -0.01)	0.002	-0.04 (-0.06; -0.02)	<0.001
Albumin/Creatinin-Ratio	0.01 (0.00; 0.02)	0.123	0.00 (-0.01; 0.01)	0.973

β-coefficients are from linear regression
